# Complications of clavicle fracture surgery in patients with concomitant chest wall injury: a retrospective study

**DOI:** 10.1186/s12891-021-04148-1

**Published:** 2021-03-20

**Authors:** Tsung-Han Yang, Huan-Jang Ko, Alban Don Wang, Wo-Jan Tseng, Wei-Tso Chia, Men-Kan Chen, Ying-Hao Su

**Affiliations:** 1 Department of Orthopedics, National Taiwan University Hospital Hsin-Chu Branch, 25, Lane 442, Sec 1, Jingguo Rd, Hsinchu City, 30059 Taiwan; 2 Department of Orthopedics, National Taiwan University Hospital, Taipei City, 10002 Taiwan; 3 Department of Surgery, National Taiwan University Hospital Hsin-Chu Branch, Hsinchu City, 30059 Taiwan; 4 Department of Emergency, National Taiwan University Hospital Hsin-Chu Branch, Hsinchu City, 30059 Taiwan; 5 Department of Biological Science and Technology, National Chiao Tung University, Hsinchu, Taiwan; 6 Department of Family Medicine, National Taiwan University Hospital Hsin-Chu Branch, Hsinchu City, 30059 Taiwan; 7 Department of Orthopedics, National Taiwan University Hospital Hsin-Chu Biomedical Park Branch, Hsinchu, County 30261 Taiwan; 8 Department of Orthopedics, National Taiwan University College of Medicine, Taipei City, 10002 Taiwan

**Keywords:** Clavicle injuries, Clavicle surgery, Hospital readmission, Postoperative complication, Rib fractures, Thoracic injuries

## Abstract

**Background:**

The impact of associated chest wall injuries (CWI) on the complications of clavicle fracture repair is unclear to date. This study aimed to investigate the complications after surgical clavicle fracture fixation in patients with and without different degrees of associated CWI.

**Methods:**

A retrospective review over a four-year period of patients who underwent clavicle fracture repair was conducted. A CWI and no-CWI group were distinguished, and the CWI group was subdivided into the minor-CWI (three or fewer rib fractures without flail chest) and complex-CWI (flail chest, four or more rib fractures) subgroup. Demographic data, classification of the clavicle fracture, number of rib fractures, and associated injuries were recorded. Overall complications included surgery-related complications and unplanned hospital readmissions. Univariate analysis and stepwise backward multivariate logistic regression were used to identify potential risk factors for complications.

**Results:**

A total of 314 patients undergoing 316 clavicle fracture operations were studied; 28.7% of patients (90/314) occurred with associated CWI. Patients with associated CWI showed a significantly higher age**,** body mass index, and number of rib fractures. The overall and surgical-related complication rate were similar between groups. Unplanned 30-day hospital readmission rates were significantly higher in the complex-CWI group (*p* = 0.02). Complex CWI and number of rib fractures were both independent factor for 30-day unplanned hospital readmission (OR 1.59, 95% CI: 1.00–2.54 and OR 1.33, 95% CI: 1.06–1.68, respectively).

**Conclusion:**

CWI did not affect surgery-related complications after clavicle fracture repair. However, complex-CWI may increase 30-day unplanned hospital readmission rates.

**Supplementary Information:**

The online version contains supplementary material available at 10.1186/s12891-021-04148-1.

## Background

Clavicle fractures have traditionally been managed with conservative treatment but in recent years surgical fixation has become increasingly popular [1–3]. Several studies have reported an improved fracture union rate with surgical treatment of displaced midshaft and distal clavicle fractures [4–9] compared to conservative treatment. The benefits of clavicle fracture surgical treatment must be balanced with the known risk of postoperative surgical complications [10].

While many clavicle fractures present as an isolated injury, a subset of patients also sustain associated injuries. Chest wall injury (CWI) is a commonly associated injury in patients sustaining a clavicle fracture with an incidence ranging from 20 to 60%, and even higher incidences have been reported in patients with open clavicle fractures [11–13]. There is a wide range in the severity of associated CWI. Concurrent rib fractures may negatively affect the stability of a fractured clavicle. Stahl et al. [14] described that concomitant ipsilateral rib fractures significantly increased the rate of displacement of unstable midshaft clavicle fractures. In severe forms of CWI, delayed and retained hemothorax or empyema may develop and impact clavicle fracture management [15–17].

To date, the impact of associated CWI on the post-operative complications in patients undergoing clavicle fracture fixation has not been thoroughly investigated. We hypothesized that CWI increases the complication rates in patients undergoing surgical clavicle fracture treatment. The purpose of this study was to compare the complication rates after surgical fixation of clavicle fractures between patients with and without CWI.

## Methods

### Study design

We performed a retrospective study using ICD-9 code 810 and ICD-10 code S42.0 to identify patients with fractures of the clavicle treated at our institution.

### Study population

Patients aged 18 years and older who were diagnosed with an acute displaced clavicle fracture and underwent surgical fixation during a 4-year period between November 2014 to October 2018 were eligible for this study. Three hundred sixty-nine clavicle fractures undergoing surgical treatment and meeting the eligibility criteria were identified. The surgical indication of all orthopedic surgeons at our institute was similar to that previously described [9, 18, 19]. Exclusion criteria were patient age < 18 years, revision fixation, and pathological fractures. Patients who were lost to follow-up before 1 year post-operatively or before bone union occurred were also excluded. Based on these criteria, we excluded 53 patients: 19 patients underwent revision surgery, 15 were < 18 years old, and 19 patients were lost to follow-up before 1 year post-operatively. This led to a study population of 314 patients with 316 clavicle fracture fixations.

Patients with an associated CWI had consultation by either a thoracic or trauma surgeon. Minimal hemothoraces were typically treated conservatively with close observation both pre- and post-operatively. For patients requiring tube thoracostomy, this procedure was typically performed in the emergency department at the time of presentation.

### Surgical technique

Twelve board-certified orthopedic surgeons in the Department of Orthopedics of our institute performed the procedures. Fractures of the clavicle were classified according to the Arbeitsgemeinschaft für Osteosynthesefragen (AO) Classification [20]. Displaced AO type 15.2 fracture were treated with either open reduction internal fixation (ORIF) with plating or intramedullary fixation with a Knowles pin. Displaced AO type 15.3 fractures were treated with ORIF using hook plates, distal clavicle locking plates, coracoclavicular stabilization, or trans-acromioclavicular fixation with a tension band wire.

All patients were treated with a standard post-operative protocol that included initiation of passive motion exercises immediately post-operatively and a sling for 4 to 6 weeks after the surgery. Patients visited the orthopedic outpatient clinic for monthly follow-ups with radiographs during the first 3 months post-operatively. Patients were typically followed for 1 year post-operatively with the timing of subsequent radiographs based on individual surgeon’s preference.

### Study procedures and variables

The demographics, body mass index (BMI), current and former smoking, presence of diabetes mellitus, laterality of rib fractures, number of rib fractures, AO classification of the fractured clavicles, and associated injuries were abstracted from the medical record. The presence of an associated injury except for chest wall injuries was defined as a concurrent injury with an Abbreviated Injury Scale ≧3 [21].

The initial chest radiograph and/or CT scan of the chest were reviewed, and diagnoses of rib fractures and associated thoracic injuries were confirmed by a thoracic and a trauma surgeon. The postoperative radiographs of the clavicle were reviewed blindly by two board-certified orthopedic surgeons (W.T.T. and Y.H.S.). Any objective radiographic complications, including fracture malunion, nonunion, implant loosening, implant breakage, implant malposition, and osteolysis, were recorded. Malunion was defined as shortening > 2 cm, angulation > 30 degree, and translation > 1 cm [22]. Nonunion was defined as no bridging callus by 6 months post-injury. Loosening was defined as presence of halo sign around implants or migration of implant compared with postoperative X-rays. Implant malposition was defined as screw violation to the acromioclavicular joint [23].

The patients were divided into a CWI group, and a no-CWI group. The CWI group was further subdivided into a minor-CWI group comprising patients who sustained one to three rib fractures but no flail chest, and a complex-CWI group comprising patients who suffered four or more rib fracture or a flail chest [24]. Flail chest was defined as at least 3 consecutive rib segmental fractures.

Dependent variables in this study included the overall complication rate, surgery-related complications, and any unplanned 30- and 90-day hospital readmissions [25]. Surgery-related complications included bone union, delayed union, implant-related, and wound complications. Wound complications comprised postoperative surgical site infections and wound dehiscence. Implant-related complications included implant breakage and malposition, loss of reduction, peri-implant fractures, delayed union and malunion, and osteolysis [26, 27]. Data on unplanned hospital readmissions were also extracted from the hospital medical records.

### Statistical analysis

The Shapiro-Wilk test was used to test the normality of variables. The data were presented as the median (interquartile range) for numerical variables and the number (percentage) for categorical variables. The Kruskal-Wallis test was used to compare numerical variables in different groups, followed by pairwise comparison by the Mann-Whitney U test. The Chi-Square test was used to compare the categorical variables. Then either the Chi-Square test or Fisher’s Exact test was used for subgroup pairwise comparison. A *p*-value < 0.05 was considered to be statistically significant. Chest wall injury was added to the models as both as a categorical variable (minor or complex CWI) and numerical variable (number of rib fractures). Stepwise backward logistic regression was used to compare independent variables and binary dependent variables. Initially, all covariates were selected for multivariate logistic regression modeling. Less significant variables (*p*-value < 0.2) were gradually eliminated from the regression model. All analyses were performed with Stata 16.0 statistical software for Windows (Stata Statistical Software, Texas, USA).

### Ethics approval

This study was approved by the Institutional Review Board of our institute with the approval number #108–005-E. Informed consent for participation in this study was waived because of its retrospective nature and the absence of any personally identifiable information.

## Results

The demographic and clinical data of the patients in the groups and subgroups are summarized in Table 1. Details of pairwise comparison in subgroup analysis was listed in Additional file 1. Ninety of the 314 enrolled patients had an associated CWI (28.5%). These patients were significantly older (*p* < 0.0001) and had a higher BMI (*p* = 0.0001). The CWI group had higher incidence of AO type 15.2 fractures (*p* = 0.041) than no CWI group (Additional file 1: Table S1). All other parameters were comparable between the groups and subgroups.

**Table 1:** Demographics and clinical characteristics of patients (*n* = 314) with displaced clavicle fractures undergoing surgeries (*n* = 316) compared between those without chest wall injury (no-CWI), concomitant CWI, and minor and complex CWI

Variable	Group				*p*-value
	No-CWI (*n* = 226)	CWI (*n* = 90)	Minor-CWI (*n* = 36)	Complex-CWI (*n* = 54)	
Age, years	40 (25–55)	53 (45–60)	53 (46–59)	53 (42–62)	**< 0.0001**
Sex, male	148 (65.5%)	56 (62.2%)	21 (58.3%)	35 (64.8%)	0.84
BMI, kg/m^2^	23 (20–26)	25 (23–27)	25 (22–27)	25 (23–27)	**0.0001**
Smoking					
Current	59 (26.1%)	31 (34.4%)	14 (38.9%)	17 (31.5%)	0.27
Previously	12 (5.3%)	1 (1.1%)	1 (2.8%)	0 (0)	0.12
Diabetes	16 (7.1%)	10 (11.1%)	4 (11.1%)	6 (11.1%)	0.57
Clavicle fracture					0.94
Left	116 (51.3%)	49 (54.4%)	20 (55.6%)	29 (53.7%)	
Right	110 (48.7%)	41 (45.6%)	16 (44.4%)	25 (46.3%)	
Rib fracture number	0 (0)	4 (2–6)	2 (1–3)	6 (5–6)	**< 0.0001**
AO type					0.088
15.1	2 (0.9%)	0 (0)	0 (0)	0 (0)	
15.2	152 (67.3%)	73 (81.1%)	28 (77.8%	45 (83.3%)	
15.3	72 (31.8%)	17 (18.9%)	8 (22.2%)	9 (16.7%)	
Associated injury	17 (7.5%)	13 (14.4%)	4 (11.1%)	9 (16.7%)	0.12

Numerical data is presented as the median (Interquartile range); categorical data as the number n (%). CWI: chest wall injury; BMI: Body Mass Index; no: Number; AO: Arbeitsgemeinschaft für Osteosynthesefragen classification. Complex CWI: flail chest, four or more rib fractures; Associated injury: abbreviated Injury Scale ≧3

Table of pairwise comparison between groups is in Additional file 1

The overall complication rate ranged from 11.1–22.2% (Table 2), and the surgery-related complication rate ranged from 5.6–12.4%. There was no significant difference in the complication rate between each group.

**Table 2:** Complications after surgical fixation based on the presence and severity of chest wall injury

Variable	Group				*p*-value
	No-CWI (*n* = 226)	CWI (*n* = 90)	Minor-CWI (*n* = 36)	Complex-CWI (*n* = 54)	
Complications					
Overall	31 (13.7%)	16 (17.8%)	4 (11.1%)	12 (22.2%)	0.35
Surgery-related	28 (12.4%)	6 (6.7%)	2 (5.6%)	4 (7.4%)	0.29
Union-related	7 (3.1%)	0 (0)	0 (0)	0 (0)	0.13
Implant-related	18 (8.0%)	5 (5.6%)	1 (2.7%)	4 (7.4%)	0.65
Wound/Infection	3 (1.3%)	1 (1.1%)	1 (2.7%)	0 (0)	0.70
Unplanned readmission					
30-day	4 (1.8%)	5 (5.6%)	0 (0)	5 (9.3%)	**0.02**
90-day	10 (4.4%)	9 (10.0%)	2 (5.6%)	7 (13.0%)	0.082

Overall complications include surgery-related complications and unplanned readmission

Union related complications included nonunion or delayed union

Subgroup analysis showed complex CWI group had significantly higher rate of unplanned 30- and 90-day readmission than no-CWI group (*p*-value = 0.005 and 0.018, respectively) (Additional file 1: Table S2)

*CWI* chest wall injury

Unplanned 30-day admission rate was increasing from the no-CWI, the CWI, to the complex CWI group (*p* = 0.02). The results of the subgroup analysis indicated that patients in the complex-CWI group had a significantly higher rate of unplanned 30- and 90-day hospital readmissions (*p* = 0.005 and 0.018, respectively) (Additional file 1: Table S2). The surgery-related complication rate was higher in the no-CWI group compared to both the minor-CWI and complex-CWI, but these differences were not statistically significant.

Stepwise backward multivariate logistic regression was performed and all covariates were selected (age, BMI, current and former smoking, diabetes, AO classification, associated injury, CWI) to identify independent risk factors for 30- and 90-day unplanned readmission (Table 3). After eliminating less significant variables (*p* < 0.2), presence of complex CWI contributed significantly to the 30-day unplanned readmission (adjusted OR 1.59, 95% CI 1.00–2.54, *p* = 0.049). Number of rib fractures contributed significantly to both the 30- and 90-day unplanned readmission (adjusted OR 1.33, 95% CI 1.06–1.68, *p* = 0.015 and adjusted OR 1.21, 95% CI 1.02–1.43, *p* = 0.028, respectively).

**Table 3:** Independent factors for unplanned readmission after surgical fixation of displaced clavicle fractures

Variable	*p*-value	Adjusted OR	95% CI
30-day unplanned readmission			
Complex CWI vs. no CWI	0.049	1.59	1.00 to 2.54
Rib fracture number	0.015	1.33	1.06 to 1.68
90-day unplanned readmission			
Complex CWI vs. no CWI	0.058	1.40	0.99 to 1.98
Rib fracture number	0.028	1.21	1.02 to 1.43

Stepwise backward multivariate logistic regression: Complex CWI (categorical) and rib fracture number (numerical) were both examined. All other covariates were selected initially. Less significant variable (*p*-value < 0.2) would be eliminated from the regression model

*OR* odds ratio, *CI* confidence interval, *CWI* chest wall injury

Nineteen patients were readmitted to hospital within 3 months of surgery. The detailed causes of these unplanned readmissions are listed in Table 4. The incidence was 4.4% (10/226) and 10% (9/90) in the no-CWI and CWI group, respectively. The surgery-related readmission rate in these patients (with unplanned hospital readmission) was 70% (7/10) and 22% (2/9) in the no-CWI and CWI group, respectively. According to the AO classification, less surgery-related complications occurred in midshaft clavicle fractures (7.1%) than in distal clavicle fractures (18%) (Table 5).

**Table 4:** Cause of unplanned readmission after surgical fixation of displaced clavicle fractures

No-CWI	n	CWI	n
Within 30 days			
Implant failure	1	Implant failure	1
Wound infection	1	Empyema	1
Post-TBI SIADH	1	Retained hemothorax	3
Urinary tract infection	1		
31–90 days			
Implant failure	4	Retained hemothorax and implant failure	1
Pneumothorax	1	Urinary tract infection	1
Wound infection	1	Posttraumatic knee stiffness	1
		Another traffic accident	1
Total	10		9

*CWI* chest wall injury, *TBI* traumatic brain injury, *SIADH* syndrome of inappropriate antidiuretic hormone secretion

**Table 5:** Complications after surgical fixation of displaced clavicle fractures differentiated by AO classification type

	AO type 15.2 Midshaft clavicle (*n* = 225)	AO type 15.3 Distal clavicle (*n* = 89)
Surgery-related complications	16 (7.1)	16 (18.0)
Nonunion/delayed union	5 (2.2)	3 (3.4)
Implant-related	8 (3.6)	12 (13.5)
Implant breakage or Loosening, malposition	7 (3.1)	5 (5.6)
Osteolysis, peri-implant fracture	1 (0.4)	7 (7.9)
Wound complications	3 (1.3)	1 (1.1)
Unplanned 90-day readmission	14 (6.2)	5 (5.6)

## Discussion

This study showed a higher unplanned hospital readmission rate in the complex-CWI group than in the no-CWI group. These patients were readmitted for the management of CWI-related pleural lesions, including subsequent retained hemothorax, or empyema. However, we found no increase in the clavicle fracture surgical complication rates in the complex-CWI group. Patients in the CWI group were significantly older than the no-CWI group, but this age difference was not found to be an independent variable in the risk for unplanned readmission.

The complications of clavicle repair in relation to the implant design and in comparison with conservative treatment have been extensively reported in the literature. However, the epidemiology of clavicle fractures with concomitant CWI and the impact of associated CWI on postoperative complications have rarely been discussed. The relationship between clavicle fracture classification and CWI incidence has been discussed by Bakir et al. [28]. They showed a significantly higher rate of concomitant thoracic injuries for medial clavicle fractures because they are located more centrally in the body, and their occurrence requires considerably more force than lateral fractures.

Bone, including those of the chest wall, becomes more brittle with age, and its mineral density decreases increasing the risk for fracture. In addition, the chondral cartilage undergoes calcification which may impact fracture risk. We found that patients with CWI were significantly older than patients with no-CWI, which may be explained due to age related changes in bone structure.

We found no significant difference in surgery-related complications between the CWI and no-CWI groups. Stahl et al. [14] described that the presence of rib fractures, especially in the upper third of the thorax, may affect the stability of conservatively treated clavicle fractures and result in unstable fractures with progressive displacement > 100% on follow-up radiographs. In our series, all patients underwent clavicle fracture repair with internal fixation, which counteracts any deforming force exerted by associated rib fractures. The associated CWI and its associated pain, may limit activity and shoulder motion in this patient group decreasing early forces on the clavicle fracture fixation. Recovery of strength and motion of the ipsilateral shoulder after CWI may require 6 to 12 weeks, even after surgical stabilization of rib fractures (SSRF) [29].

The results of the subgroup analysis indicated that patients in the complex-CWI group had a significantly higher rate of unplanned 30- and 90-day hospital readmission. Complex-CWI patients had intrapleural lesion including retained and delayed hemothorax and empyema [30]. Other conditions that have been reported in complex CWI include a trapped lung or diaphragm laceration [31, 32]. Our study showed that the management of these CWI-related intrapleural complications was the major cause of unplanned hospital readmissions.

Unplanned 30-day readmission is a parameter commonly used as a quality of hospital care metric [25]. In our cohort, complex-CWI was an independent risk factor for 30-day unplanned readmission. Subsequent complications, such as delayed and retained hemothorax and empyema, often occur within 1 month of the initial trauma. Our analysis of the causes of 90-day unplanned readmissions showed that revision for failed clavicle surgery or the management of complications related to associated injuries was more frequent than for 30-day readmissions. Complex CWI did not independently impact on the 90-day unplanned readmission rate.

The majority of 30-day unplanned hospital readmissions were due to complex CWI-related complications. Therefore, the appropriate management of complex CWI may be an important step to reduce unnecessary readmission. In recent years, some studies have reported that SSRF in complex CWI provides short-term benefits that are superior to conservative treatment [33–36]. Furthermore, early SSRF may provide several advantages over late SSRF [37–39]. Zhang et al. [40] stated that a dedicated thoracic trauma team with multidisciplinary medical professionals might be necessary to reduce postoperative complication of patients with complex CWI. However, the indications for SSRF are still unclear, and no guidelines exist for the use of SSRF in patients without respiratory failure. More evidence is required to guide the optimal treatment for complex CWI.

There are several advantages of this study. This study demonstrated that concomitant CWI may have a negative impact on the outcome of clavicle fracture repair. Presence of complex CWI and number of rib fractures are both independent risk factor of 30-day unplanned readmission and most of the causes of unplanned readmission is to manage complex CWI-related intrapleural lesion. Minor CWI is not an independent risk factor of unplanned readmission. The rate of surgery-related complication is similar in patients with and without CWI.

Our results have to be interpreted within the limitation of this study. This was a retrospective single-center study in a relatively small study population. CT scan of the chest was not done in patients with only one or two rib fractures. We did not consider subjective complications, such as functional scores or skin paresthesia, which are considered as complications of clavicle fracture repair by some authors. We further did neither assess the relationship between different implants and implant- related complications nor the use of narcotics in this study [41].

Clavicle fracture repair is generally safe in patients with both minor CWI and complex CWI. However, if a patient sustains a complex CWI, surgeons should be aware of the potential thoracic complications such as subsequent hemothorax, retained hemothorax, or empyema which may present on a delayed basis and require unplanned hospital readmission.

## Conclusion

In patients undergoing surgical clavicle fracture repair, concomitant CWI did not affect surgery-related complications. Those with concomitant complex-CWI, may result in a higher rate of unplanned 30- and 90-day readmissions. Further studies are required to improve the quality of clavicle fracture repair and reduce the complications following clavicle fracture surgery in patients with concomitant CWI.

## Supplementary Information

**Additional file 1: Table S1.** Pairwise comparison of demographics and clinical characteristics of patients (*n* = 314) with displaced clavicle fractures undergoing surgeries (*n* = 316) compared between those without chest wall injury (CWI), concomitant CWI, and uncomplicated and complex CWI. **Table S2.** Pairwise comparison of complications after surgical fixation based on the presence and severity of chest wall injury.

## Data Availability

The data are available from the corresponding author upon reasonable request.

## Abbreviations

CWI: Chest wall injury
AO: Arbeitsgemeinschaft für Osteosynthesefragen
ORIF: open reduction internal fixation
BMI: body mass index
SSRF: surgical stabilization of rib fractures.
