# Supplementary material for: Complications of clavicle fracture surgery in patients with concomitant chest wall injury: a retrospective study
Source: BMC Musculoskelet Disord. 2021 Mar 20;22:294. doi: 10.1186/s12891-021-04148-1 (PMC7981946; doi:10.1186/s12891-021-04148-1)
Supplement: Supplementary file 1 — Additional file 1: Table S1. Pairwise comparison of demographics and clinical characteristics of patients (n = 314) with displaced clavicle fractures undergoing surgeries (n = 316) compared between those without chest wall injury (CWI), concomitant CWI, and uncomplicated and complex CWI. Table S2. Pairwise comparison of complications after surgical fixation based on the presence and severity of chest wall injury. [file 12891_2021_4148_MOESM1_ESM.docx]

**Table S1.**  Pairwise comparison of demographics and clinical characteristics of patients (n = 314) with displaced clavicle fractures undergoing surgeries (n = 316) compared between those without chest wall injury (CWI), concomitant CWI, and uncomplicated and complex CWI

| Variable | Group | | | |  | *p*-value | | |
| --- | --- | --- | --- | --- | --- | --- | --- | --- |
|  | No-CWI  (n = 226) | CWI  (n = 90) | Minor CWI  (n = 36) | Complex CWI  (n = 54) |  | No-CWI  vs. CWI | No-CWI  vs. minor CWI | No-CWI  vs. complex CWI |
| Age, years | 40 (25–55) | 53 (45–60) | 53 (46–59) | 53 (42–62) |  | **<0.0001** | **0.0002** | **<0.0001** |
| Sex, male, n(%) | 148 (65.5) | 56 (62.2) | 21 (58.3) | 35 (64.8) |  | 0.58 | 0.41 | 0.95 |
| BMI, kg/m^2^ | 23 (20–26) | 25 (23–27) | 25 (22–27) | 25 (23–27) |  | **0.0002** | 0.054 | **0.0003** |
| Smoking, n(%) |  |  |  |  |  |  |  |  |
| Current | 59 (26.1) | 31 (34.4) | 14 (38.9) | 17 (31.5) |  | 0.14 | 0.11 | 0.43 |
| Previously | 12 (5.3) | 1 (1.1) | 1 (2.8) | 0 (0) |  | 0.09 | 0.52 | 0.08 |
| Diabetes | 16 (7.1) | 10 (11.1) | 4 (11.1) | 6 (11.1) |  | 0.24 | 0.40 | 0.32 |
| Clavicle fracture, n(%) |  |  |  |  |  | 0.62 | 0.84 | 1 |
| Left | 116 (51.3) | 49 (54.4) | 20 (55.6) | 29 (53.7) |  |  |  |  |
| Right | 110 (48.7) | 41 (45.6) | 16 (44.4) | 25 (46.3) |  |  |  |  |
| Rib fracture number | 0 (0) | 4 (2-6) | 2 (1-3) | 6 (5-6) |  | **<0.0001** | **<0.0001** | **<0.0001** |
| AO type |  |  |  |  |  | **0.041** | 0.41 | 0.062 |
| 15.1 | 2 (0.9) | 0 (0) | 0 (0) | 0 (0) |  |  |  |  |
| 15.2 | 152 (67.3) | 73 (81.1) | 28 (77.8) | 45 (83.3) |  |  |  |  |
| 15.3 | 72 (31.8) | 17 (18.9) | 8 (22.2) | 9 (16.7) |  |  |  |  |
| Associated injury | 17 (7.5) | 13 (14.4) | 4 (11.1) | 9 (16.7) |  | 0.059 | 0.46 | **0.038** |
| Numerical data is presented as the median (Interquartile range); categorical data as the number n (%). CWI: chest wall injury; BMI: Body Mass Index; no: Number; AO: Arbeitsgemeinschaft für Osteosynthesefragen classification. Complex CWI: flail chest, four or more rib fractures; Associated injury: abbreviated Injury Scale ≧3. | | | | | | | | |

**Table S2**. Pairwise comparison of complications after surgical fixation based on the presence and severity of chest wall injury.

| Variable | Group | | | |  | *p*-value | | |
| --- | --- | --- | --- | --- | --- | --- | --- | --- |
|  | No-CWI  (n = 226) | CWI  (n = 90) | Minor CWI  (n = 36) | Complex CWI  (n = 54) |  | No-CWI  vs. CWI | No-CWI  vs. minor CWI | No-CWI  vs. complex CWI |
| Complications, n(%) |  |  |  |  |  |  |  |  |
| Overall | 31 (13.7) | 16 (17.8) | 4 (11.1) | 12 (22.2) |  | 0.36 | 0.67 | 0.12 |
| Surgery-related | 28 (12.4) | 6 (6.7) | 2 (5.6) | 4 (7.4) |  | 0.14 | 0.23 | 0.30 |
| Union-related | 7 (3.1) | 0 (0) | 0 (0) | 0 (0) |  | 0.20* | 0.60* | 0.35* |
| Implant-related | 18 (8.0) | 5 (5.6) | 1 (2.7) | 4 (7.4) |  | 0.46 | 0.27 | 0.89 |
| Wound/Infection | 3 (1.3) | 1 (1.1) | 1 (2.7) | 0 (0) |  | 1* | 0.45* | 1* |
| Unplanned readmission, n(%) | |  |  |  |  |  |  |  |
| 30-day | 4 (1.8) | 5 (5.6) | 0 (0) | 5 (9.3) |  | 0.068 | 1.00* | **0.005** |
| 90-day | 10 (4.4) | 9 (10.0) | 2 (5.6) | 7 (13.0) |  | 0.060 | 0.67* | **0.018** |

CWI: chest wall injury; vs: versus

Overall complications include surgery-related complication and unplanned readmission.

*Fisher’s Exact test was used.
